# Radiosensitizing Effect of Gold Nanoparticles Stabilized with Sodium Citrate and Thioglycolic Acid on MDA-MB-231 and MCF7 Breast Cancer Cells

**DOI:** 10.3390/ijms27198632

**Published:** 2026-09-26

**Authors:** Anna Smirnova, Elizaveta Kontareva, Zain Nofal, Valery Kryuchikhin, Anna Chigasova, Nikita Drozhzhin, Evgeny Andreev, Irina Fadeikina, Alisher Mutali, Alexander Nechaev, Sergey Leonov, Andreyan N. Osipov, Margarita Pustovalova

**Affiliations:** 1 Institute of Future Biophysics, Moscow Center for Advanced Studies, Moscow 123592, Russia; smirnova.av@phystech.edu (A.S.); montag_sever@mail.ru (E.K.); zainnufal@gmail.com (Z.N.); annagrekhova1@gmail.com (A.C.); sergey.leonov@yahoo.com (S.L.); pu.margo@mail.ru (M.P.); 2 State Research Center-Burnasyan Federal Medical Biophysical Center of Federal Medical Biological Agency, Moscow 123098, Russia; oval33423@mail.ru; 3 Emanuel Institute of Biochemical Physics of RAS, Moscow 119334, Russia; 4 Joint Institute for Nuclear Research, Dubna 141980, Russia; dna.17@uni-dubna.ru (N.D.); evandreev@jinr.ru (E.A.); i.fadeikina@yandex.ru (I.F.); mutali@jinr.ru (A.M.); nechaeffalexander@yandex.ru (A.N.); 5 Faculty of Natural Sciences and Engineering, Dubna State University, Dubna 141980, Russia; 6 Laboratory of Solid State Physics, Institute of Nuclear Physics, Almaty 050032, Kazakhstan; 7 Institute of Cell Biophysics of Russian Academy of Sciences, Pushchino 142290, Russia; 8 CANDLE Synchrotron Research Institute, 31 Acharyan, Yerevan 0040, Armenia

**Keywords:** nanoparticles, radiosensitization, DNA damage response (DDR), breast cancer

## Abstract

Gold nanoparticles (AuNP) have been increasingly investigated as potential radiosensitizing agents for the treatment of cancer. In the present study, AuNP stabilized with sodium citrate and thioglycolic acid (AuNP-Cit-TGA) were synthesized to assess their radiosensitizing effects on MDA-MB-231 and MCF7 breast cancer cells after X-ray exposure. The AuNP-Cit-TGA displayed an average diameter of 22 nm, showcasing extremely low hemolytic activity in vitro, along with a moderate rate of cellular uptake. AuNP-Cit-TGA notably decreased cell viability in both cell lines 48 h after exposure to a radiation dose of 6 Gy, as demonstrated by the Alamar Blue assay. Dose–response clonogenic assay indicated a significant decrease in the survival of nanoparticle-treated MCF7 cells compared to control groups. This decrease was associated with heightened oxidative stress, as evidenced by increased DCFH fluorescence and a decrease in the expression of *SOD2*, *GPx1*, *CAT*, and *TXRDN1* genes 24 h following 2 Gy exposure. Remarkably, the uptake of AuNP-Cit-TGA enhanced the occurrence of single-strand DNA breaks in MCF7 cells by nearly double when compared to radiation treatment alone. However, this effect was not statistically significant in MDA-MB-231 cells. Despite this, residual γH2AX and 53BP1 foci were observed in both cell lines, revealing ongoing unrepaired DNA double-strand breaks connected to the uptake of nanoparticles by cells. Collectively, these findings provide valuable insights into the application of AuNP-Cit-TGA as an effective radiosensitizing agent in breast cancer radiotherapy.

## 1. Introduction

Breast cancer (BC) is the second most commonly diagnosed cancer globally, with about 2.3 million new cases and approximately 670,000 deaths annually worldwide (https://www.iarc.who.int/ accessed on 1 March 2026). Current data indicates that every minute, four women are diagnosed with BC globally, and one woman dies from the disease. If trends continue, by 2050 there could be 3.2 million new cases and 1.1 million deaths each year from BC worldwide. Multiple genomic assays have been increasingly employed to direct chemotherapy decision-making for patients with early-stage BC, whereas radiation therapy (RT) has traditionally been applied as a neoadjuvant, adjuvant, or palliative modality following a ‘one-size-fits-all’ paradigm [1]. RT is inherently limited by the imperative to restrict radiation dose in order to minimize collateral damage to surrounding healthy tissues. However, such dose constraints may inadequately control tumor cells exhibiting radioresistance, thereby potentially diminishing therapeutic efficacy. To address these challenges, contemporary research has focused on leveraging genomic biomarkers, circulating tumor material, quantitative imaging metrics, the tumor microenvironment, and the microbiome to refine and personalize RT strategies. Furthermore, to reduce the incidence of long-term adverse effects, including normal tissue toxicity, the development and implementation of nanoparticle (NP)-based radiosensitizers have been actively pursued.

High-Z elements absorb energy better than soft tissues and release secondary photons and electrons (such as photoelectrons and Compton electrons) when exposed to ionizing radiation [2]. Secondary electrons damage DNA and react with water, leading to the formation of reactive oxygen species (ROS), which heighten tumor cell sensitivity to radiation [3]. Therefore, nanomaterials made of high-Z elements act as radiosensitizers, reducing the dosage needed to eliminate tumors and lowering the risk of harmful side effects. Gold is a high-Z element (Au, Z = 79) and gold NPs have emerged as a promising tool for cancer diagnosis and treatment due to their chemical inertness, strong photoelectric interaction, high surface-to-volume ratio, and biocompatibility [4]. Additionally, their tumor-targeting capabilities through the enhanced permeability and retention (EPR) effect, along with their versatility in disease diagnosis and bioimaging, further enhance their therapeutic potential [5,6,7,8]. Multiple formulations of gold NPs, their particle size and morphology, surface characteristics, concentration, as well as biodistribution and specific localization, affect the effectiveness of gold NPs in radiotherapy [4,9,10]. Sodium citrate and thioglycolic acid used as a reducing and capping agents with gold NPs proved to have very good biocompatibility and good cytotoxicity against cancer cells [11,12]. But their combined effect has not been previously elucidated on the radiosensitization properties of gold NPs against cancer cells, especially BC with different metastatic potential.

Our recent studies have shown that cellular biophysical traits like stiffness might play a significant role in cellular sensitivity to radiation. Moreover, this radiosensitivity varies significantly among BC cell lines, with the MDA-MB-231 triple-negative BC (TNBC) cells being substantially more radiosensitive than the MCF7 (estrogen receptor positive, ER+) cells [13]. Notably, MDA-MB-231 cells possess highly-metastatic properties [14] and efficiently uptake NPs [15], supporting the promise of NP-based radiosensitizers as a targeted therapeutic approach for the eradication of this aggressive cell phenotype.

In this study, we evaluated the effectiveness of a radiosensitizing strategy using gold NPs stabilized with sodium citrate and thioglycolic acid (AuNP-Cit-TGA) on BC cells with different genomic profiles and metastatic potential by comparing their effects on TNBC MDA-MB-231 and ER+ MCF7 cell lines.

## 2. Results

### 2.1. Characterization of NPs

Figure 1a presents the absorption spectrum of gold NPs stabilized with sodium citrate and thioglycolic acid (AuNP-Cit-TGA), exhibiting a plasmon resonance peak at 535 nm, characteristic of gold NPs. The average diameter of AuNP-Cit-TGA particles was 20 ± 5 nm as determined by transmission electron microscopy (TEM). A histogram of NPs size distribution and TEM images is presented in Figure 1b,c. The polydispersity index (PDI) for NPs was 0.06, which indicates the monodispersity of the sample. The shape of the NPs was not perfectly round as the images show clumps or overlaps of many NPs. Furthermore, the average zeta potential of the particles was −25 mV at pH = 7, reflecting their stability.

For subsequent experiments, the synthesized gold NPs were purified and concentrated on an ultrafilter using a tangential filtration system. The AuNP-Cit-TGA concentration was 1300 μg/mL, and the pH was adjusted to 7.

To evaluate the hydrodynamic size of AuNP-Cit-TGA, the particle size distribution histogram obtained via DLS in water and DMEM medium after incubation for 24 h at 37 °C (Figure 2) was studied.

Size distribution histograms present an increase of the AuNP-Cit-TGA hydrodynamic size in DMEM medium compared to water, namely, from 41 ± 17 nm in water up to 82 ± 39 nm in DMEM. No significant changes in hydrodynamic size after incubation during 24 h at 37 °C in water were observed. The hydrodynamic size increased by 10 nm after incubation for 24 h at 37 °C in DMEM medium.

### 2.2. Biocompatibility of Synthesized NPs

The hemolytic activity of NPs at a final concentration of 0.5, 5, 25, 50, 100 and 200 μg/mL was analyzed by human erythrocyte lysis assay. The degree of lysis was assessed by measuring the optical density at 540 nm, which indicates the level of hemoglobin released. This was compared to the positive control (100% lysis in H_2_O) and the negative control (0% lysis in PBS). Incubation with AuNP-Cit-TGA resulted in a remarkably low hemolysis value, with optical density measurements comparable to the negative control levels (Figure 3a). The mean hemolysis values for each concentration of NPs for four individual donors are presented in Table 1. These results highlight low acute in vitro toxicity toward blood components, as they are well under the ASTM E2524-08 standard threshold of 5% for hemolysis.

The Alamar Blue assay was used to study AuNP-Cit-TGA NP-induced changes in metabolic activity in human peripheral blood mononuclear cells (PBMC). Our results indicate that incubation of PBMC with NPs at a final concentration of 0.5, 5, 25, 50, 100 and 200 μg/mL led to an insignificant decrease in lymphocyte viability by 0.8, 1.6, 8.1, 4.6, 6.4 and 7.4%, respectively (Figure 3b).

### 2.3. Cellular Uptake of NPs

The localization of AuNP-Cit-TGA near the cell surface was studied using SEM. A combination of backscattered and secondary electron (BSE and SE) detectors allows visualization of NPs near the surface and the external morphology of cells. Additionally, EDS allows confirmation of the composition (Au) within the regions of interest. Figure 4a,b shows micrographs of MCF7 and MDA-MB-231 cells with the highest detected amounts of AuNP-Cit-TGA, as well as EDS-spectra of the regions containing NPs.

Micrographs of MCF-7 cells treated with AuNP-Cit-TGA show small, bright particulate regions, whereas micrographs of MDA-MB-231 cells exposed to NPs display larger bright areas. EDS analysis identifies these signals as gold. Notably, the particles visible in the images appear to lie slightly above the cell surface: SE images show no marked changes in cell topography, and attempts to focus on the cell membrane produce a blurring of the NPs.

ICP-OES was used to evaluate the uptake of NPs between 1 h and 24 h of incubation (Figure 4c). Extending the NPs incubation time to 24 h resulted in a 2.5% increase in intracellular NPs content in MCF7 cells and a 1.9% increase in MDA-MB-231 cells. Thus, extending the incubation period to 24 h produced only a slight increase in intracellular NP accumulation in both cell lines, suggesting that NP uptake was largely achieved during the earlier incubation period. The effect was somewhat more pronounced in MCF7 cells than in MDA-MB-231 cells. Thus, a 1 h incubation period was sufficient for cellular uptake of the NPs, which is consistent with previous studies reporting a more rapid cellular uptake of NPs smaller than 30 nm [16,17].

NP uptake was additionally evaluated using flow cytometry. Analysis of side scattering (SSC) intensity reflects the internal granularity of the cells. Internalization of NPs resulted in increases of cells’ granularity and subsequently their SSC without changing the forward scatter (FSC) intensity. We analyzed the changes in the SSC channel after incubating MCF7 and MDA-MB-231 cells with NPs at a concentration of 0.5 μg/mL. The proportion of cells with SSC intensities exceeding the reference intensity was measured (i.e., “% of cells with high SSC”, the reference intensity was defined as the mean SSC value + 1 standard deviation”) (Figure 4d). MDA-MB-231 and MCF7 cells demonstrated 25.6% and 20.3% of cells encapsulating NPs (“SSC-A+ cells, %”), respectively (Figure 4e). These values exceeded the control MCF7 and MDA-MB-231 cells’ values by 3.9% (non-significant, *p* > 0.05) and 9% (*p* < 0.05), respectively. According to flow cytometry data, MDA-MB-231 cells encapsulate AuNP-Cit-TGA NPs more effectively than MCF7 cells.

### 2.4. NP’s Impact on Cell Metabolic Activity After Irradiation at a Dose of 6 Gy

Cell metabolic activity was assessed 48 h after exposure to 6 Gy irradiation using the Alamar Blue assay. Based on our previous findings demonstrating the biological effectiveness of gold NPs at 0.5 μg/mL, this concentration was selected for the present study. Treatment with NPs significantly reduced the metabolic activity of both non-irradiated MCF7 and MDA-MB-231 cells by 20% (*p* < 0.05 and *p* < 0.01, respectively) relative to untreated control groups (Figure 5a,b). Upon irradiation at 6 Gy, NPs exhibited modest cytotoxic effects, decreasing cell metabolic activity to 70.5% for both MCF7 (*p* < 0.01) and MDA-MB-231 cells (*p* < 0.001), as assessed by Alamar Blue staining, compared to untreated controls (Figure 5a). Furthermore, pre-treatment with AuNP-Cit-TGA prior to irradiation increased cytotoxic effects by factors of 1.3 (*p* < 0.01) and 1.2 in MCF7 and MDA-MB-231 cells, respectively, compared to irradiation alone.

We used the Bliss independence method to see if irradiation and adding NPs to the cells worked together in any way [18]. The Bliss independence framework was chosen because it does not require obtaining the observable effect over a range of doses, which is ideal when using one dose of irradiation in combination with one concentration of NPs. In case of the Alamar Blue measurements for MCF7 cells, the observed effects of NPs and IR alone were 0.203 and 0.066, giving an expected combined effect of 0.256 under independence, while the observed effect of the combination was 0.295 (EBliss = +0.04). For MDA-MB-231, the corresponding single-agent effects were 0.212 and 0.141, giving an expected value of 0.324 versus an observed value of 0.295 (EBliss = −0.03). Neither deviation from Bliss independence was statistically significant (*p* = 0.56 and *p* = 0.76, respectively), indicating no detectable synergy under these conditions.

### 2.5. NP-Mediated Oxidative Stress Analysis

Mitochondria are both a major source of ROS and a target for ROS generated by high-Z metal NPs in cancer cells. The Alamar Blue test evaluates mitochondrial function by analyzing the change of resazurin into resorufin. This is indicated by a strong shift in color and light. Therefore, we expected an increase of ROS production upon the decrease of cell metabolic activity assessed by the Alamar Blue assay. Thus, we evaluated ROS production in cells by measuring DCFH fluorescence 24 h after exposing them to 2 Gy X-rays. AuNP-Cit-TGA NPs themselves increased ROS production by 2 times and 1.7 times in unexposed MCF7 (*p* < 0.05, Figure 6a) and MDA-MB-231 (*p* < 0.05, Figure 6b) cells, respectively. NPs increased ROS generation by 2 times (*p* < 0.001) in MCF7 and 1.6 times (*p* < 0.01) in MDA-MB-231 when followed by 2 Gy irradiation, compared to irradiated controls without NPs. When compared to untreated controls, these values were more than 3 times higher in both cell lines (*p* < 0.0001). The results suggest that using AuNP-Cit-TGA NPs with irradiation could effectively cause oxidative stress in BC cells.

### 2.6. NP’s Impact on Single-Strand DNA Breaks Formation

High-Z metal NPsmake cells more sensitive to radiation by creating ROS, which lead to breaks in DNA strands [19]. Genotoxicity of AuNP-Cit-TGA and the ability to enhance the formation of radiation-induced single-strand breaks (SSBs) were assessed by comet assay, as published previously [20]. The movement of DNA, like the tail section and its distance, is related to SSBs and weak points in cells, as seen in Figure 7a.

In MCF7 cells, treatment with NPs led to a two times higher tail DNA (%) than in control cells (*p* < 0.05), whereas in MDA-MB-231 cells, the damaged DNA level did not change (Figure 7b). NPs also exhibited a significant radiosensitizing effect on MCF7 cells, leading to a two-fold increase in the proportion of single-strand breaks compared to irradiation alone (*p* < 0.0001). In MDA-MB-231 cells, the highest percentage of damaged DNA was seen in the NPs + IR group rather than in the cells that were irradiated without NPs, but this difference was not significant. Overall, AuNP-Cit-TGA markedly increased the yield of radiation-induced DNA SSBs in BC cells, causing 38% more damage in MCF7 cells (*p* < 0.0001) and 24% more damage in MDA-MB-231 cells (*p* < 0.0001) compared with control cells.

### 2.7. NP’s Impact on Antioxidant Gene Expression

To grasp the difference between the uptake of NPs (Figure 4) and level of SSBs, we analyzed *SOD1*, *SOD2*, *GPx1*, *TXNRD1*, *TXNRD2* and *CAT* genes expression levels by PCR 24 h after 2 Gy irradiation (Figure 8). MDA-MB-231 cells showed a generally stronger antioxidant gene expression profile than MCF7 cells, especially for *GPx1*, which is expressed at a dramatically higher level in MDA-MB-231 under all conditions. *SOD1*, *SOD2*, and *CAT* were also higher in MDA-MB-231 than in MCF7, while *TXNRD1*, and *TXNRD2* showed more modest, gene-specific, and treatment-dependent changes between the two lines.

NPs treatment alone induced an increase in *SOD1* and *CAT* expression—by 1.5-fold and 1.25-fold in MCF7 and MDA-MB-231 cells, respectively—compared tocontrol cells. More pronounced differences were observed for *SOD2*. In MCF7 cells, its expression was 3-fold upregulated following NPs treatment, whereas in MDA-MB-231 cells it increased 1.8 fold compared with the control level. In contrast, *TXNRD1* and *TXNRD2* expression decreased in both cell lines under the same conditions. No significant differences in gene expression were observed between the IR and IR + NPs groups, with the exception of *CAT* and *TXNRD2* expression in MCF7 cells (1.3-fold increase, *p* < 0.001 and *p* < 0.05, respectively). The unchanged key parts of the antioxidant system in MDA-MB-231 cells may show a possibly lower level of ROS compared to MCF7 cells 24 h following 2 Gy exposure. This, in turn, correlates with a reduced proportion of DNA SSBs in MDA-MB-231 cells. Overall, irradiation and AuNP-Cit-TGA exposure modulate these genes in a cell line–dependent manner, with the strongest and most consistent distinction observed for *GPx1*.

### 2.8. NP’s Impact on DNA Repair Response

DNA double-strand (DSBs) breaks are the most lethal type of DNA damage. The efficient repair of DSBs is crucial in maintaining genomic integrity and cellular viability [21]. Analysis of phosphorylated H2AX (γH2AX) foci acts as a sign of an early step in the DSB response. This leads to changes in chromatin structure at the damaged area to help DNA repair. The p53-binding protein 1 (53BP1) is vital for repairing DNA breaks. It promotes non-homologous end-joining (NHEJ) repair while inhibiting homologous recombination (HR) repair [22]. These DNA repair foci accumulate, peaking 0.5–1 h after IR exposure [23], and then degrade exponentially to almost control levels. Having residual foci 24 h after exposure is associated with reduced cell survival and growth [21]. This suggests that DNA damage is still present, possibly leading to cell death or aging [24]. Hence, we next assessed γH2AX and 53BP1 foci numbers in NPs-treated cells 1 h and 24 h after exposure to 2 Gy irradiation (Figure 9).

There was no NP-induced increase in the number of γH2AX foci in MCF7 cells at 1 h after irradiation (NPs + IR vs. IR group, Figure 9a, left panel). Compared to control (without NPs, IR group), NPs-treated cells had significantly higher residual foci numbers at 24 h after 2 Gy exposure (*p* < 0.01). In MDA-MB-231 cells, incubating with NPs for 1 h significantly increased γH2AX foci (control vs. NPs, Figure 9a, right panel). Irradiating NP-treated cells leads to a minimal increase in γH2AX foci compared to irradiated (IR only) cells without NPs. The presence of residual γH2AX foci was characteristic of these cells, as they were unable to reach the level of control (IR only) cells 24 h after irradiation (*p* < 0.001) (Figure 9a, right panel).

Treatment with NPs resulted in statistically significant lower numbers of 53BP1 foci in 2 Gy irradiated MCF7 cells (NP + IR, *p* < 0.0001) compared to 2 Gy irradiated cells without NPs (IR only, Figure 9b left panel). The residual 53BP1 foci numbers were much higher in NP-treated cells (NP + IR) than in untreated cells and NP-treated cells at 24 h after radiation, indicating unrepaired DNA damage. A slight decrease in the number of 53PB1 foci was also noticed in irradiated NP-treated MDA-MB-231 cells (Figure 9b right panel). The highest number of residual radiation-induced 53BP1 foci at 24 h post-radiation wasobserved in cells treated with AuNP-Cit-TGA (NP + IR) (*p* < 0.001 and *p* < 0.0001 compared to NPs only and IR group, respectively). Collectively, our results demonstrate that AuNP-Cit-TGA NPs were effective in causing DNA damage in breast cancer cells 24 h after exposure to 2 Gy X-rays.

### 2.9. NP’s Impact on Clonogenic Growth

We evaluated the radiosensitization effect of NPs using clonogenic assay. Survival enhanced ratio (SER) was calculated for 37% survival (SF = 0.37) (Figure 10). In MCF7 cells, the 37% surviving fraction was reached at approximately 3.1 Gy for the control group and 2.3 Gy for the NPs group, based on the estimation from the survival curves (Figure 10a). This corresponds to anSER of 1.35, testifying to a modest radiosensitization effect of NPs. In MDA-MB-231 cells, the dose required to achieve 37% survival was 2.2 Gy in the both NP-treated and control groups, corresponding to an estimated SER of 1 (Figure 10b). Despite this, cells treated with NPs exhibited a steeper curve than the control, although the differences did not reach statistical significance.

Comparison of the α/β ratios (Table 2) showed that MCF7 cells treated with NPs exhibited an increased α/β ratio (3.88 Gy) compared to cells without NPs (2.95 Gy), a marked reduction in the shoulder of the survival curve and a shift toward more linear, less repairable radiation damage. This change is consistent with NP-induced radiosensitization, whereby cells become less capable of repairing sublethal damage and more prone to lethal damage per unit dose. Similar effects were observed for MDA-MB-231 cells, where the addition of NPs resulted in a more pronounced α/β ratio (3.45 Gy) compared to cells without NPs (0.24 Gy). Collectively, these results indicate that NPs exert a potential radiosensitization effect, as evidenced by cell type-related viability declines of BC cell lines.

## 3. Discussion

Recent advances in nanomedicine increasingly emphasize multifunctional and precision-oriented platforms that combine therapeutic activity with diagnostic monitoring. Nanoengineered systems can be designed to promote tumor-selective oxidative stress, catalytic therapy, photothermal effects, or enhanced cellular delivery, while nanoprobe technologies—including colloidal gold, quantum dots, magnetic nanoparticles, nanozymes, aggregation-induced-emission materials, and upconversion NPs—enable sensitive biomarker detection and treatment monitoring. The emerging integration of such nanoplatforms with artificial-intelligence-enabled biomedical engineering may further support data-driven optimization of NP composition, targeting, safety, and individualized therapeutic response [23,25,26,27,28]. In this study, gold NPs stabilized with sodium citrate and thioglycolic acid (AuNP-Cit-TGA) were synthesized to assess their effectiveness as radiosensitizers for BC cells during X-ray irradiation. Our synthesized NPs demonstrated favorable in vitro biocompatibility and moderate internalization by BC cells. We recently demonstrated that MDA-MB-231 cells with high metastatic potential encapsulate synthetic NPs more efficiently than low metastatic MCF7 cells [15,29]. Our present results further support this notion, showing that MDA-MB-231 more efficiently internalized AuNP-Cit-TGA NPs than MCF7 cells (Figure 4). This ability can be linked to the stiffness of the cell membranes and nucleus, with MDA-MB-231 cells known to be softer than MCF7 cells [13]. The stiffness of the nucleus affects the compaction of heterochromatin and the ability of cells to form DNA repair complexes [14,30]. However, this inherent “softness” did not affect the overall radiosensitizing efficacy of the AuNP-Cit-TGA in MDA-MB-231 cells compared to “stiffer” MCF7 cells.

MCF7 cells exhibited overall greater NP-based radiosensitivity, producing more ROS (Figure 6) that amplify DNA SSBs (Figure 7) and DSBs (Figure 8). The baseline level of DNA SSBs in MCF7 cells (~8%) is notably higher than in MDA-MB-231 cells (~2%) (Figure 7b). Following irradiation at a dose of 2 Gy, the level of DNA damage becomes comparable in both cell lines (~22–23%). However, upon the addition of NPs, MDA-MB-231 cells demonstrate resistance to further damage, whereas in MCF7 cells, DNA damage increases to approximately 50% after irradiation in the presence of NPs. A similar pattern is observed in the clonogenic assay performed across irradiation doses of 2, 4, and 6 Gy, both with and without NPs. In MCF7 cells, NPs induced a statistically significant decrease in survival (colony formation) compared to the control, while no significant differences between NP-treated and untreated groups were observed in MDA-MB-231 cells (Figure 9b).

The observed difference in radiosensitivity between MCF7 and MDA-MB-231 cells appears to be explained by differences in the basal expression of antioxidant defense genes. The most pronounced difference was observed for *GPx1* (glutathione peroxidase 1, which reduces H_2_O_2_ to water using glutathione). Its expression in MDA-MB-231 cells was ~35-fold higher than in MCF7 (Figure 8), consistent with reports of epigenetic silencing of *GPX1* in luminal ERα-positive cell lines due to promoter methylation [31]. Higher GPx1 expression in MDA-MB-231 cells [32] might help eliminate NP-induced ROS more effectively. This could result in fewer DNA SSBs, as indicated by the comet assay (Figure 7a,b). In MCF-7 cells, low GPx1 expression in combination with elevated intracellular ROS levels may serve as biochemical indicators of ferroptosis [33,34]. *SOD1* (Cu/Zn superoxide dismutase, which converts superoxide to H_2_O_2_) and *CAT* genes (which decomposes H_2_O_2_ into water and oxygen independently of glutathione) were also significantly more highly expressed in MDA-MB-231, supporting this interpretation. The *TXNRD1* gene (cytosolic thioredoxin reductase, which supports peroxiredoxins and DNA repair via ribonucleotide reductase) did not differ significantly between the cell lines. It was down-regulated in response to AuNP-Cit-TGA exposure, which is consistent with previous studies where encapsulation of gold NPs correlated with inhibition of TXNRD1 [35]. If TXNRD1 is low, MDA-MB-231 cells do not survive well and are more affected by radiation [36]. Our results demonstrate that exposure to radiation restores TXNRD1 activity. Thus, the elevated levels of *GPx1*, *SOD1*, and *CAT* in MDA-MB-231 likely account for its lower baseline DNA damage and resistance to additional NP-induced sensitization, whereas the lack of such antioxidant protection in MCF7 appears to underlie its increased radiosensitivity and the more pronounced radiosensitizing effect of NPs in this cell line.

Gold NPs are emerging novel radiosensitizers for cancer patients due to their unique properties [4,37,38]. Gold NPs show synthetic versatility, unique chemical, electronic, and optical properties, and high X-ray absorption [39]. Their catalytic activity facilitates oxidative stress, which is a key mechanism underlying NP-induced cellular toxicity [36]. Furthermore, the high atomic number (high-Z) of gold (Au, Z = 79), enhances the radiosensitization of cells through the formation of secondary photons and electrons upon irradiation [2]. This leads to an increased intracellular ROS yield and subsequently induces extensive DNA damage, predominantly SSBs and oxidized DNA bases [35].

Potentially, NPs entering the nucleus can enhance DNA damage. Since the inner nuclear pore width is 40–60 nm, our 22 nm gold NPs might be able to access the nucleus. Moreover, upon internalization, NPs can be transported to other cellular organelles, such as mitochondria. However, intra-organelle delivery of NPs requires a positive zeta potential, which is not the case in our study [40]. Overall, the synergistic effect of gold NPs and irradiation increases the level of DNA damage in BC cells. Thus, our results are consistent with earlier studies that found gold NPs lead to greater DNA damage and reduced clonogenic growth [41].

Research indicates that the size, surface modifications of NPs, and incubation time before irradiation significantly influence their radiosensitizing effect [42,43]. Nanomaterials elicit cellular responses that depend on the functional groups present on their surfaces, which can be either specific (e.g., particular chemical molecules) or non-specific (e.g., simple functional groups like –OH or –COOH). The chemical properties of nanomaterials, like their elemental makeup, unique biochemical molecules, and metal ion release, mainly determine their toxic effects on the body.

Sodium citrate acts as a reducing agent and stabilizer in the synthesis of gold NPs [44,45]. Citrate ions reduce gold ions to metallic gold and create a negatively charged layer that prevents aggregation, helping to maintain the stability of the colloidal suspension [46,47]. Thioglycolic acid (TGA) consisting of a thiol (−SH) group, acts as a capping agent since thiol groups are able to spontaneously form a stable Au–S dative bond. Several studies suggest using TGA-capped gold NPs as biosensors [48,49,50,51]. However, the combined contribution of sodium citrate and TGA to the radiosensitizing properties of gold NPs in BC cells has not been previously elucidated.

For the first time, gold NPs stabilized with sodium citrate and thioglycolic acid (AuNP-Cit-TGA) were studied for their effects on the survival and γH2AX and 53BP1 foci formation in MCF7 and MDA-MB-231 BC cells after X-ray exposure. The NPs formulation and concentration used in this study effectively increased radiosensitization in MCF7 BC cell lines.

## 4. Materials and Methods

### 4.1. Synthesis Methodology

The synthesis of gold nanoparticles stabilized with sodium citrate and thioglycolic acid (AuNP-Cit-TGA) was carried out based on [48]. For this purpose, 40 mL of deionized water (Milli-Q, Millipore, Burlington, MA, USA, with a resistivity of 18 MΩ cm at 22 °C) were heated to 80 °C, after which 88 μL of a 113 mM solution of H[AuCl_4_]. HAuCl_4_∙3H_2_O (≥49.0% (Au), Acros Organics, Geel, Belgium) was used to prepare the solution, and after 3 min, 1 mL of a solution containing 0.0089 g of sodium citrate was added (sodium citrate 5.5-aqueous, 98%, Panreac (Barcelona, Spain) was used to prepare the solution). Heating was continued for 15 min with stirring, then cooled in a water bath to room temperature. After cooling the nanoparticle solution, a thioglycolic acid solution was added (10 μL of thioglycolic acid (98%, Acros Organics, Geel, Belgium) in 5 mL of water with pH = 10, obtained using 0.1 M NaOH), and left with stirring for 30 min. Nanoparticle samples were purified on a polysulfone ultrafilter (100 kDa) using a preparative tangential filtration system developed at the Flerov Laboratory of Nuclear Reactions of the Joint Institute for Nuclear Research. A 5-roller peristaltic pump (Heidolph, Schwabach, Germany) was used to deliver the solution. The solution flow rate was 2.6 mL/s, the cell inlet pressure was 1.5 bar, and the linear flow velocity near the membrane surface was 3.2 m/s. The samples were concentrated from 200 mL to 9 mL four times, with intermediate dilutions of the concentrate.

### 4.2. Characterization of NPs

To characterize the shape and size distribution of NPs, transmission electron microscopy (Thermo Scientific, Waltham, MA, USA, Talos F200iS/TEM) was used. Copper grids with a thin amorphous carbon film (SPI supplies) served as substrates. For nanoparticle deposition, the substrate was immersed in the colloidal solution of gold NPs, then air-dried. Using the JMicroVision 1.3.4 software, the nanoparticle sizes and standard deviation were calculated based on the micrographs. For each sample, an analysis of a sample set including at least 500 particles were conducted. PDI was calculated according to the following formula:

(1) $$PDI=\frac{\sigma }{d}$$

where σ—standard deviation (nm), d —average diameter (nm)

The concentrations of elements were analyzed on an Expec 6000 (Thermo Scientific, Waltham, MA, USA) inductively coupled plasma atomic emission spectrometer using the calibration method. The optical properties and changes in the concentration of NPs were determined using the absorption spectra in the ultraviolet and visible regions obtained on a dual-beam spectrophotometer Evolution 600 (Thermo Scientific, Waltham, MA, USA) with an optical path length of 1 cm based on [52]. The zeta potential of the synthesized NPs was measured by laser Doppler microelectrophoresis using a Zetasizer Nano ZSP instrument (Malvern Panalytical Ltd., Malvern, UK). A U-shaped cuvette with built-in gold electrodes DTS1070 (Malvern Panalytical Ltd., Malvern, UK) was used for the measurements.

### 4.3. Evaluation of Compatibility and Biosafety of Synthesized NPs Using Erythrocyte Hemolysis Assay

Blood compatibility was evaluated with hemolytic activity of NPs on human red blood cells. Fresh blood was obtained from healthy donors. 2 mL of whole blood sample was mixed with 4 mL of Dulbecco’s phosphate-buffered saline (D-PBS, Paneco, Moscow, Russia) and then centrifuged at 3000 *g* for 5 min to isolate red blood cells. The red blood cells were then washed five times with 10 mL of D-PBS. 0.18 mL of the diluted red blood cell suspension was mixed with 0.02 mL of the nanoparticle suspension at a final concentration of NPs 0.5, 5, 25, 50, 100 and 200 μg/mL. The addition of distilled water and D-PBS (Paneco, Moscow, Russia) were used as the positive (100% lysis) and negative (0% lysis) groups, respectively. After incubation at 37 °C for 4 h, the samples were centrifuged for 10 min at 3000 RPM, 100 μL of the supernatant of all samples were transferred to a 96-well plate and the absorbance was measured using a CLARIOstar microplate reader (BMG LABTECH, Ortenberg, Germany) at 540 nm. The degree of hemolysis was expressed by the hemolytic ratio according to the following formula:

(2) $$Hemolysis\;ratio=\frac{\left(OD(test)-OD(negative\;control)\right)}{\left(OD(positive\;control)-OD(negative\;control)\right)}\times 100\%$$

### 4.4. Alamar Blue Assay of Cell Viability

Lymphocytes were isolated from fresh human blood using a Ficoll (Paneco, Moscow, Russia) gradient. The lymphocyte suspension diluted in RPMI1640 medium was added to the wells of a 96-well plate at a concentration of 1 × 10^5^ cells/100 μL of suspension/well. NPs were added to a final concentration of 0.5, 5, 25, 50, 100, and 200 μg/mL and incubated for 24 h at 37 °C in a CO_2_ incubator.

To assess the viability of the BC cells, they were seeded in 96-well plates at a density of 1 × 10^4^ cells per well. The following day, NPs were administered 1 h prior to irradiation. Subsequently, the cells were incubated for 48 h at 37 °C in a humidified atmosphere containing 5% CO_2_.

After incubation period, alamarBlue™ (Cell Viability Reagent, Invitrogen, Carlsbad, CA, USA, cat. No. DAL1100) was added in a volume of 1/10 of the total volume of the medium in the wells and incubated for 3 h at 37 °C and 5% CO_2_. Fluorescence intensity was analyzed using a CLARIOstar tablet multifunctional analyzer (BMG LABTECH, Ortenberg, Germany) at 560/580 nm wavelength. Relative cell viability was calculated using the following formula:

(3) $$Cell\;viability=\frac{Fluorescence\;intensity\;of\;treated\;cells}{Fluorescence\;intensity\;of\;control\;cells}\times 100\%$$

### 4.5. Irradiation

The cells were irradiated using a RUB RUST-M1 (JSC “Ruselectronics”, Moscow, Russia) X-ray biological setup equipped with two X-ray emitters at a dose rate of 0.85 Gy/min, a voltage of 200 kV, a current of 2 × 5 mA, and a 1.5 mm Al filter. An ionization dosimeter DRK-1-K01 (SPC Doza, Moscow, Russia) and GAFChromic EBT3 dosimetry film (Ashland Advanced Materials, Bridgewater, NJ, USA) were used to estimate the absorbed dose. The total uncertainty of the dispensed absorbed dose did not exceed 15%.

### 4.6. Cell Cultures

The human BC cell lines MDA-MB-231 (ER-/PR-/EGFR+/HER2-) and MCF7 (ER+/PR+/EGFR-/HER2-) obtained from the ATCC were used in the work. The cells were cultured in DMEM medium (Gibco, Waltham, MA, USA) supplemented with 10% fetal bovine serum, L-glutamine, and 1% penicillin/streptomycin (Sigma-Aldrich, St. Louis, MO, USA) at 37° C and 5% CO_2_.

### 4.7. Analysis of Nanoparticle Localization Using Scanning Electron Microscopy

Silicon substrates were cleaned by immersion in 35% hydrogen peroxide (34.5–36.5%, Sigma-Aldrich) for 120 min, then rinsed sequentially in deionized water and isopropanol (99.9%, Sigma-Aldrich). All solutions were prepared with deionized water (Milli-Q, Millipore, Burlington, MA, USA; 18 MΩ·cm at 22 °C). Cells were incubated with nanoparticles for 24 h, harvested by trypsinization, washed, and fixed in 4% paraformaldehyde for 15 min. A 3 µL aliquot of the fixed cell suspension was applied to each silicon substrate and allowed to sit for 15 min. Excess liquid was removed and the substrates were rinsed with 50 µL deionized water. Samples were dehydrated through a graded isopropanol series (10, 30, 50, 70, and 100%), 10 min per step, then air-dried for 2 h and further dried in a vacuum oven (Binder, Neckarsulm, Germany) at 100 mbar for 12 h. Prior to imaging, a 5 nm chromium coating was sputtered onto the samples (Q150T S, Quorum, Laughton, UK) to improve surface conductivity. Scanning electron microscopy with energy-dispersive X-ray spectroscopy (EDS) was carried out on a Hitachi S-3400N microscope (Tokyo, Japan) operated at 15 kV.

### 4.8. Analysis of Nanoparticle Uptake Using ICP-OES

Cells were incubated with NPs for 1 h or 24 h and then collected by trypsinization, washed, and fixed in 4% paraformaldehyde for 15 min. Before analysis, cellular suspensions were centrifuged at 300 *g* for 5 min with following removal of supernatant. Then, 6 mL of water were added, and centrifugation procedure was repeated to remove buffer residues. Finally, 2 mL of water were added and the number of cells was counted. Next, 900 µL of each sample was transferred to a drying oven (Binder, Neckarsulm, Germany) and incubated at 80 °C overnight. Next, 200 µL of aqua regia (for the evaluation of Au uptake) or nitric acid (for the evaluation of Ag uptake) was added to the dry sediments and the resulting solutions were immersed in a sonication bath (Bandelin, Berlin, Germany, 35 kHz) for 5 h at 80 °C. After sonication, the samples were diluted with water to a final volume of 2 mL. The concentrations of elements were analyzed on an Expec 6000 (Thermo Scientific, Waltham, MA, USA) inductively coupled plasma atomic emission spectrometer using the calibration method.

### 4.9. Analysis of Nanoparticle Uptake by Breast Cancer Cells Using Flow Cytometry

Nanoparticle cellular uptake analysis was performed according to the protocol described previously [53,54]. Cells were seeded in 6-well plates. The next day, NPs were added at a final concentration of 0.5 μg/mL for 24 h. Cells were incubated at 37 °C and 5% CO_2_ and then fixed with 4% paraformaldehyde for 15 min at room temperature. Cells were analyzed using CytoFLEX™ flow cytometer (Beckman Coulter, Brea, CA, USA). The proportion of SSC+ cells was evaluated using FlowJo v.10.9.0 software (Becton, Dickinson & Company, Ashland, OR, USA).

### 4.10. DCFDA/H2DCFDA Cellular Reactive Oxygen Species (ROS) Assay

The 5000 cells per well were seeded in 384-well plates. The next day, AuNP-Cit-TGA at a concentration of 0.5 µg/mL were added to cells followed by 2 Gy irradiation after 1 h. After incubation for another 24 h, the culture media were removed, and cells were washed with PBS once. Cells were incubated with 10 µM solution of 2′,7′-Dichlorofluorescin Diacetate DCFH-DA (C6827, Invitrogen™, Thermo Fisher, Eugene, OR, USA) in a culture medium in the absence of serum for 45 min at 37 °C and 5% CO_2_. After that, the cells were washed with PBS (Paneco, Moscow, Russia) once. A solution of complete phenol red free culture medium was added to the wells and the cells were analyzed using a CLARIOstar multifunctional analyzer (BMG LABTECH, Ortenberg, Germany) at a wavelength of 485/535 nm. Data were normalized to correspondent controls without 2′,7′-Dichlorofluorescin Diacetate DCFH-DA and calculated as the ratio of fluorescence in cells treated with NPs over unirradiated controls without NPs.

### 4.11. Antioxidant Gene Expression Level Analysis

RNA extraction was performed using the phenol–chloroform method. Upon isolation, the RNA quantity was measured using the NanoDrop spectrophotometer (Thermo Fischer Scientific, Rockford, IL, USA). Furthermore, the RNA integrity was determined by performing RNA Agarose Gel Electrophoresis using 1% agarose (1 g/100 mL) and 1 × TAE buffer supplemented with 20 uL of Ethidium bromide to enable image visualization using the ChemiDoc MP Imaging System (170–8280) by Bio-Rad (Hercules, CA, USA).

Subsequently, the RNA extracts were treated with DNAse reagent using (DNase I, RNase-free) kit by (Thermo Scientific™, Rockford, IL, USA) following the manufacture’s guideline to remove any possible DNA contamination. This was then followed up by RT-qPCR to quantify the expression levels of the target genes (*SOD2*, *TXNRD1*, *CAT*, *GPx1* and *GAPDH* (reference gene)) using BioMaster RT-qPCR SYBR Blue (2×) kit (Biolabmix, Novosibirsk, Russia). The primer sequences were as follows:

*SOD1–*F–CTCACTCTCAGGAGACCATTGC

*SOD1*–R–CCACAAGCCAAACGACTTCCAG

*SOD2*–F–CTGGACAAACCTCAGCCCTAAC

*SOD2*–R–AACCTGAGCCTTGGACACCAAC

*TXNRD1–*F–GTTACTTGGGCATCCCTGGTGA

*TXNRD1*–R–CGCACTCCAAAGCGACATAGGA

*TXNRD2–*F–TTGAGGTCTATCACGCCC

*TXNRD2*–R–CTTGAGTAACTTCGCCTGC

*CAT–*F–GTGCGGAGATTCAACACTGCCA

*CAT–*R–CGGCAATGTTCTCACACAGACG

*GPx1*–F–GTGCTCGGCTTCCCGTGCAAC

*GPx1*–R–CTCGAAGAGCATGAAGTTGGGC

To ensure the accuracy and reproducibility of the results, appropriate controls were included in each RT-qPCR experiment, including non-template controls and reference genes for normalization purposes. Data analysis was performed using the Delta Ct Method (2-ddCT) in (Bio-rad CFX Maestro Software v. 3.1), and gene expression levels were calculated relative to the reference gene.

### 4.12. Immunofluorescent Analysis of γH2AX and 53BP1 foci

The cells were seeded in a 96-well plate at a concentration of 10^4^ cells/well. NPs were added at a final concentration of 0.5 µg/mL. Cells without NPs were used as a control. One hour and 24 h after irradiation at a dose of 2 Gy, the cells were fixed with 4% paraformaldehyde for 15 min at room temperature and permeabilized for 40 min at room temperature in 0.3% Triton X-100 solution with the addition of 2% bovine serum albumin. The cells were incubated for one hour with primary anti-rabbit antibodies to γH2AX (dilution 1:200, clone EP854(2)Y, Merck–Millipore, Burlington, MA, USA) and anti-mouse antibodies to 53BP1 (dilution 1:200, Cat. # MAB3802, Merck–Millipore, Burlington, MA, USA). After washing off unbound antibodies, a solution of secondary antibodies Alexa Fluor^®^ 488 (Goat Anti-Rabbit IgG H&L (Alexa Fluor^®^ 488), 1:2000 dilution, Cat. #AP132JA4, MilliporeSigma™, Burlington, MA, USA) and goat anti-mouse antibodies conjugated with Cy3 (1:500 dilution; Cat. #AP124C, Merck–Millipore, Burlington, MA, USA) were added for 1 h at room temperature. After washing, nuclei were stained with Hoechst 33,342 dye (Life Technologies Corporation, Carlsbad, CA, USA). Cells were visualized on an EVOS M5000 microscope (Invitrogen, Carlsbad, CA, USA) with a 40 x objective. Foci were counted manually.

### 4.13. Comet Assay for DNA Damage Analysis

After incubation with NPs, cells were detached using TrypLE™ Express (Life Technologies Corporation, Carlsbad, CA, USA). Single cell suspensions were mixed with low melting point agarose and spread onto microscope slides precoated with normal melting point agarose, forming a thin agarose gel layer with embedded cells. After solidification for 10 min in +4° C, cells were covered with cell culture medium and irradiated at 2 Gy X-rays. Immediately after irradiation, slides were transferred into lysis buffer (2.5 M NaCl, 100 mM EDTA, 20 mM Tris–HCl, pH 10.0, 1% Triton X-100, 10% DMSO) for 1 h. Slides were then placed in an electrophoresis chamber with an alkaline solution (300 mM NaOH, 1 mM EDTA pH > 13.0) to denature DNA and allow the expression of single-strand breaks, alkali-labile sites, and incomplete excision repair sites. The electrophoresis was run in the alkaline buffer at 0.75 V/cm and 41 C for 20 min. The samples were then neutralized to reconstitute the DNA via three rinses in 0.4 M Tris–HCl buffer, pH 7.4. The slides were slightly dried and fixed (70% ethanol for 10 min). For DNA staining, SybrGreen I (Invitrogen, Carlsbad, CA, USA) was used. Visualization of DNA comets was carried out using a fluorescence microscope Nikon Eclipse Ni-U microscope (Nikon, Tokyo, Japan) equipped with a high-definition camera ProgRes MFcool (Jenoptik AG, Jena, Germany). On each slide, 100 comets were recorded. For analysis and processing of microphotographs of DNA comets, the CASP 1.2.2 program (CASPlab) was used. As a criterion of DNA damage, the Olive tail moment was used (the product of the percentage of DNA in the tail and the distance from the center of the nucleus to the center of DNA density in the tail of the DNA comet in pixels).

### 4.14. Clonogenic Assay

Cells were seeded into 6 well plates at a density of 3 × 10^5^ cells per well, and NPs were added 1 h before irradiation. After irradiation, cells were trypsinized, counted, and seeded into 6-well plates at appropriate densities for each dose (100, 500, 1000, and 2000 cells/well for 0, 2, 4, and 6 Gy, respectively). Untreated cells were used as a control to determine plating efficiency (PE). Cells were cultured under standard conditions for 21 days to allow colony formation, then fixed with methanol and stained with Giemsa. Colonies were counted manually. Dose–response curves were generated from the mean of three independent experiments, each biological replicate comprising at least three technical replicates. Plating efficiency (PE) and survival fraction (SF) were calculated as follows:

(4) $$PE=\frac{number\;of\;colonies}{number\;of\;cells\;seeded\;in\;a\;well}\times 100\%$$

(5) $$SF=\frac{number\;of\;colonies\;formed\;after\;irradiation}{number\;of\;cells\;seeded\;in\;a\;well}\times PE$$

Sensitizer enhancement ratio (SER) was used for quantifying how much nanoparticles increase cell sensitivity to radiation using the following equation:

(6) $$SER=\frac{D0}{Ds}$$

where *D*0 gives a 37% survival level without NPs and DS is the same survival level in the same experiment with NPs.

### 4.15. Synergy Definition

We used the Bliss independence method to qualify and quantify the expected synergetic, additive, or null response of administering NPs together with irradiation in BC cells. EBliss represents the Bliss excess (or combination index), which measures the deviation of an observed effect from the expected effect using the following equation:

(7) EBliss = E_NPs + IR_ − (E_NPs_ + E_IR_ − E_NPs_ × E_IR_)

where E_NPs+IR_ is the observed effect of the NPs + IR combination and E_NPs_ and E_IR_ are the observed individual effects of NPs and IR, respectively. EBliss = 0 is the threshold for additivity, while EBliss > 0 indicates synergy and EBliss < 0 indicates antagonism.

### 4.16. Statistical Analysis

Statistical analysis was performed using Welch’s *t*-test and the Mann–Whitney U Test using GraphPad Prism 8 software (GraphPad Software, San Diego, CA, USA). Normality was estimated using Shapiro–Wilk test. Results are presented as means ± standard deviations from four independent experiments.

## 5. Limitations of Current Study

NPs had a narrow radiosensitization effect, which could affect its relevance in biology and clinical settings given the conditions tested, since we examined only a few types of radiation doses, NP concentrations, and incubation periods. These findings might be limited to the specific MCF7 and MDA-MB-231 BC cell lines. They have different genetic backgrounds, DNA repair abilities, and antioxidant status, making it hard to generalize. The investigated endpoints were assessed at different time points and therefore do not represent a single cellular state. Although these time points were selected to reflect the expected kinetics of DNA damage, oxidative stress, DNA repair responses, and subsequent cell death, the design does not allow definitive conclusions about the temporal or causal relationships between these processes. Further time-course experiments using common sampling intervals and additional mechanistic approaches are required to validate the proposed sequence of events.

Therapeutic NP concentrations achievable in tumors in vivo are typically much lower than in vitro due to EPR limitations (tumor heterogeneity, different vascular density, ECM stiffness), blood compatibility, and off-target sequestration, making cell-based “non-toxic” effects not fully translatable into human safety. While our results provide good support for the biocompatibility and safety of synthesized NPs for intravenous injection, most NPs can accumulate in the liver and spleen, leading to potential organ toxicity. Hence, in vivo experiments of NPs distribution warrants further investigation to ensure our observed in vitro NP toxicity is fully translatable to humans.

Nevertheless, our study provides, for the first time, the potential of using gold NPs stabilized with sodium citrate and thioglycolic acid as radiosensitizers against BC cells with different metastatic potential.

## 6. Conclusions

Collectively, this study demonstrates that NPs stabilized with sodium citrate and thioglycolic acid (AuNP-Cit-TGA) act as effective radiosensitizers in ER+/PR+ (MCF7) but not in triple-negative (MDA-MB-231) breast cancer cells. In MCF7 line, the 22 nm particles reduced cell viability 48 h after a 6 Gy X-ray exposure and significantly suppressed clonogenic survival. Radiosensitization was associated with elevated oxidative stress, reflected by increased DCFH fluorescence and reduced expression of the antioxidant genes *SOD2, GPx1, CAT*, and *TXNRD1* 24 h after irradiation. The NPs also nearly doubled the incidence of single-strand DNA breaks in MCF7 cells relative to radiation alone, while the persistence of unrepaired γH2AX and 53BP1 foci in both cell lines indicates the induction of long-lived DNA double-strand breaks. Taken together with the favorable hemocompatibility and moderate cellular uptake, these results support the further evaluation of AuNP-Cit-TGA as a promising radiosensitizing agent for breast cancer radiotherapy.

## Figures and Tables

**Figure 1:**
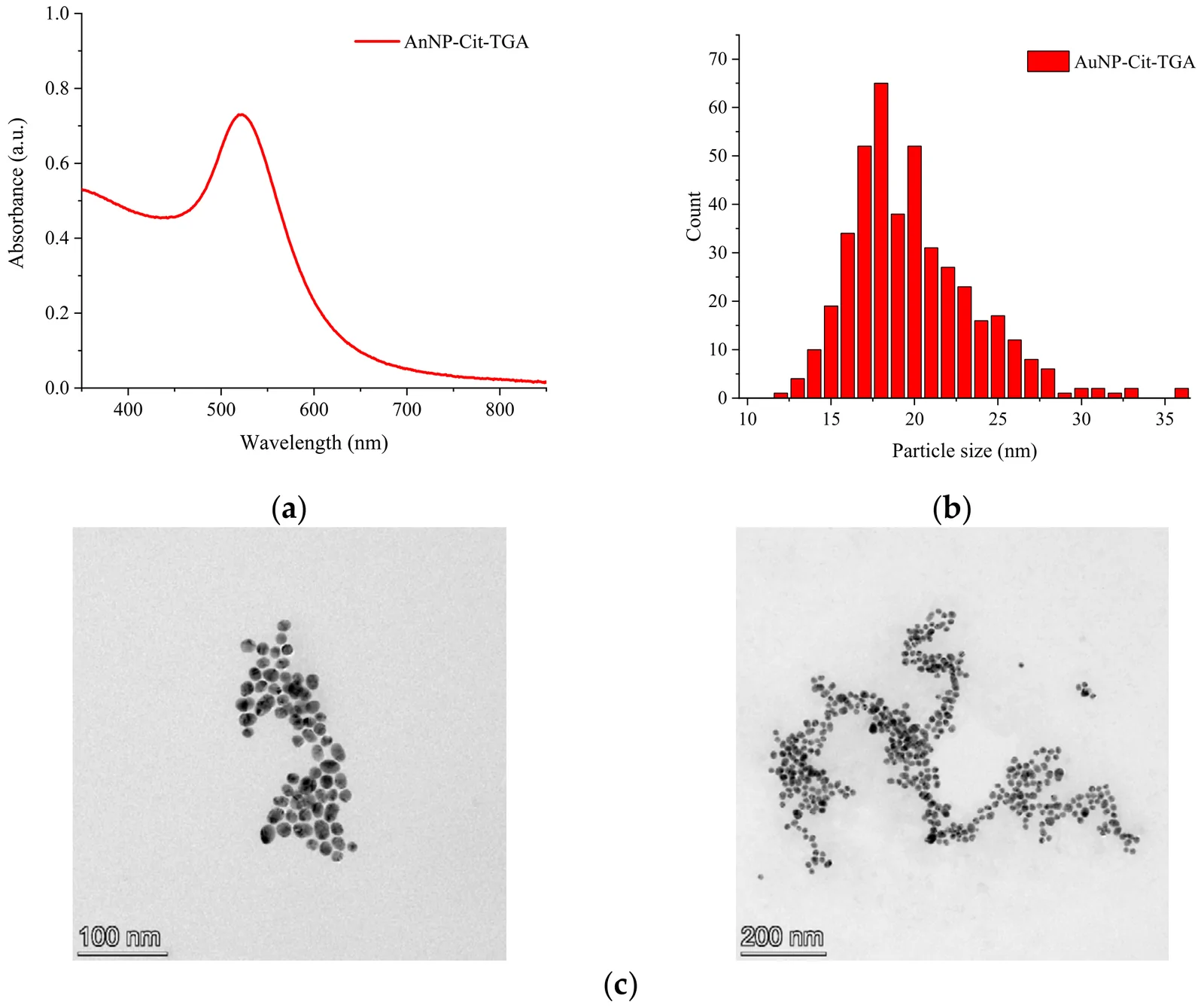
Characterization of gold NPs stabilized with sodium citrate and thiolycolic acid (AuNP-Cit-TGA). (**a**) UV-visible absorption spectrum, (**b**) histogram of AuNP-Cit-TGA size distribution, (**c**) transmission electron micrographs of AuNP-Cit-TGA. Scale bars are 100 nm (left) and 200 nm (right).

**Figure 2:**
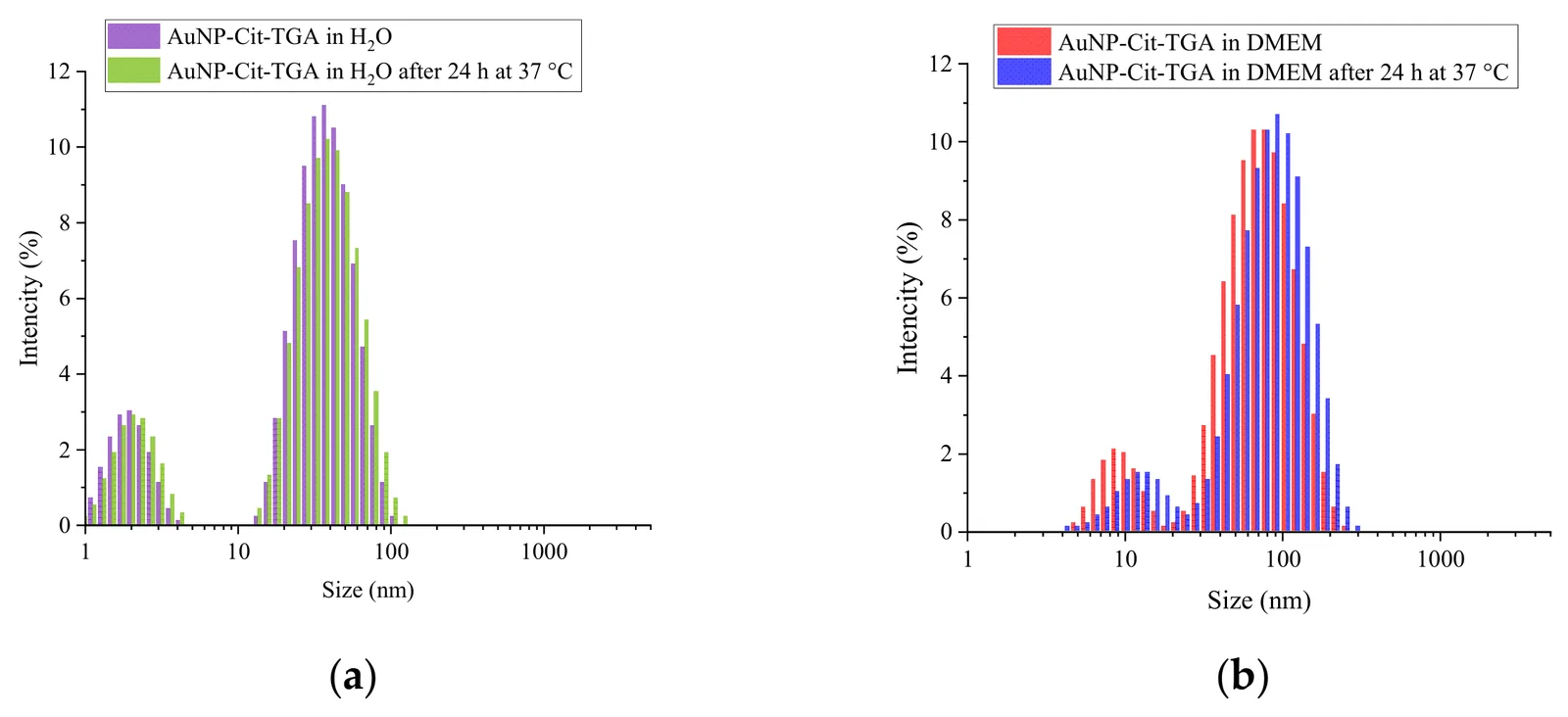
Particle size distribution histograms for AuNP-Cit-TGA obtained via DLS in water (**a**) and DMEM (**b**) medium after incubation during 24 h at 37 °C.

**Figure 3:**
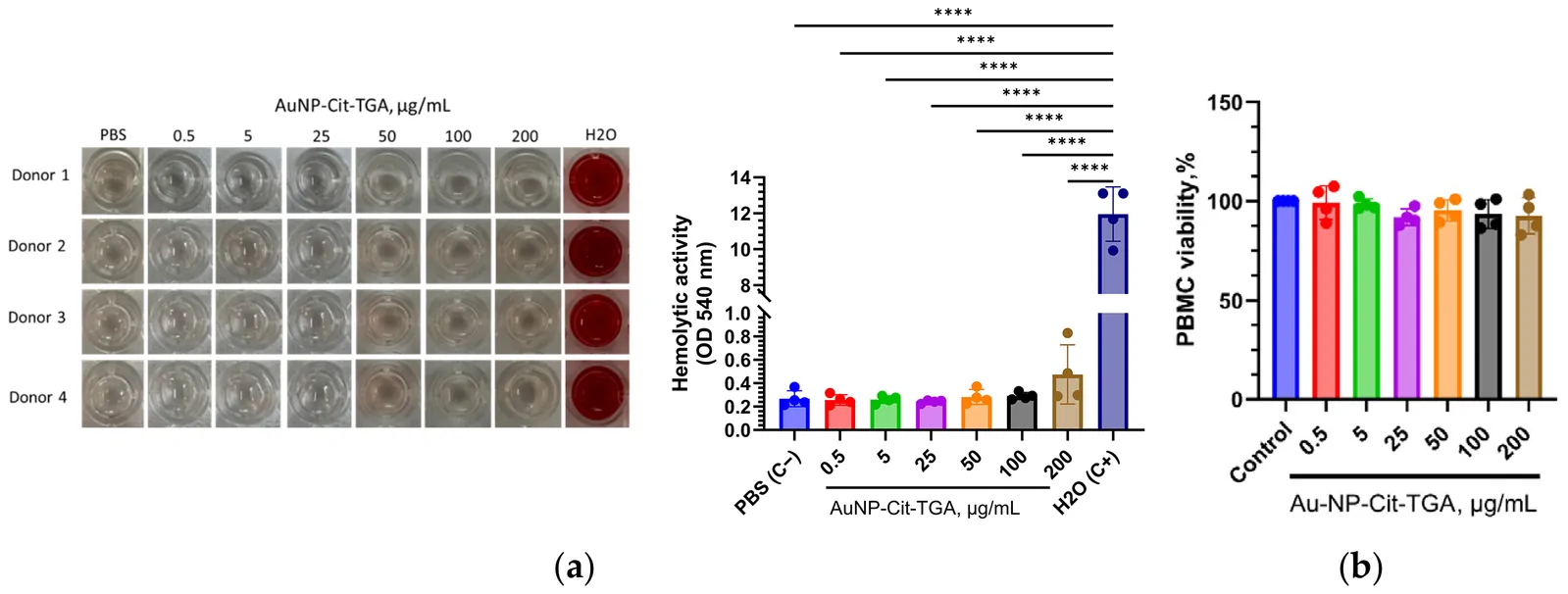
The biocompatibility of AuNP-Cit-TGA NPs and metabolic activity of PBMCs using erythrocyte hemolysis and Alamar Blue assays, respectively. (**a**) Representative photographs of erythrocytes hemolysis after 4 h of co-incubation with NPs at final concentrations of 0.5, 5, 25, 50, 100 and 200 μg/mL and the optical density of supernatant (540 nm). (**b**) The proportion of viable PBMCs following 24 h co-incubation with NPs at final concentration of 0.5, 5, 25, 50, 100 and 200 μg/mL. Data are means ± SD from four healthy donors. Each donor sample was tested in triplicates. **** *p* < 0.0001 by one-way ANOVA. Normality was tested using the Shapiro–Wilk test.

**Figure 4:**
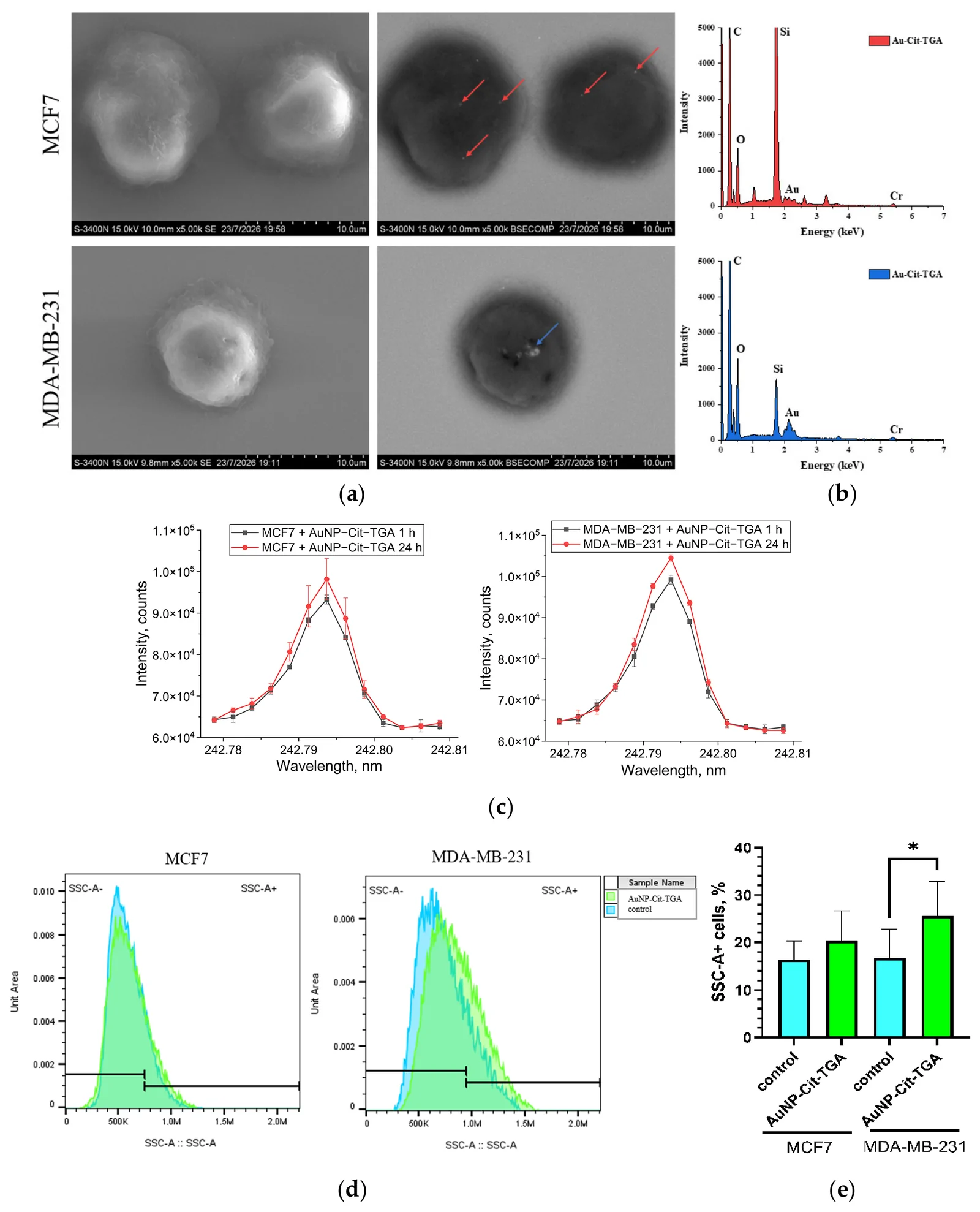
Analysis of NPs uptake by MCF7 and MDA-MB-231 cells. (**a**) Micrographs of NP localization obtained by SEM in SE and BSE detectors. (**b**) Energy-dispersive spectra of the marked spots. (**c**) ICP-OES analysis of NPs uptake after 1 h and 24 h of incubation with BC cells. (**d**) Flow cytometric analysis of NPs uptake by BC cell lines. SSC histograms and (**e**) the proportion of cells encapsulated NPs. Cells encapsulating NPs were defined as “SSC-A+” ((**b**), black histograms) population or “SSC-A+ cells, %“ ((**c**), black bars). The reference intensity was defined as “mean SSC + 1 standard deviation”. Data are means ± SD from 4 independent experiments. * *p* < 0.05 by Mann–Whitney U test.

**Figure 5:**
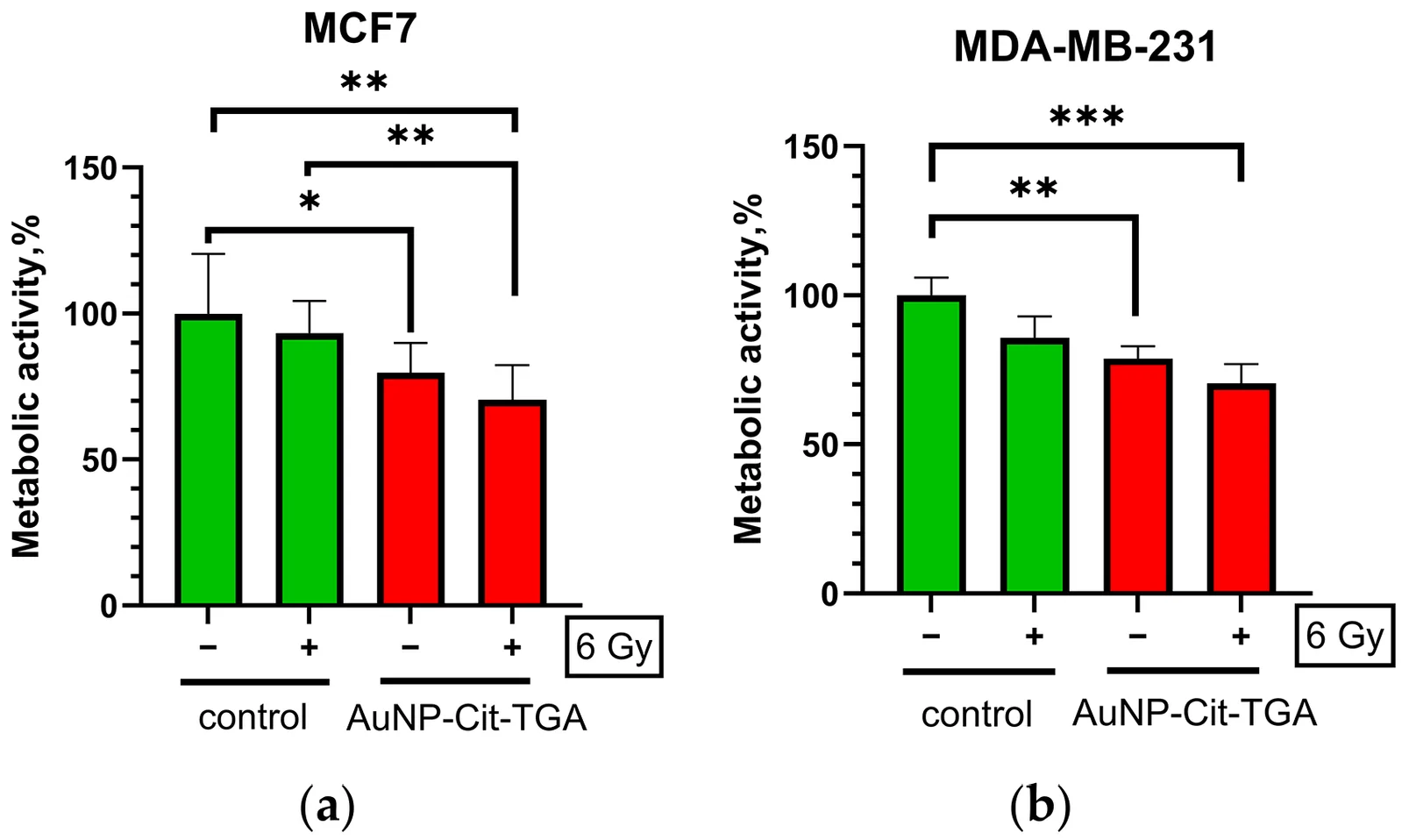
Metabolic activity of (**a**) MCF7 and (**b**) MDA-MB-231 cells in response to radiation and pre-treatment with NPs. Cells were co-incubated with NPs for 1 h before irradiation at final concentration of 0.5 μg/mL. Metabolic activity was assessed by Alamar Blue assay at 48 h after either no (-) or 6 Gy (+) irradiation. Data a means ± SD from 4 independent experiments. Data normalized to untreated controls. * *p* < 0.05; ** *p* < 0.01, *** *p* < 0.001 by Mann–Whitney U test.

**Figure 6:**
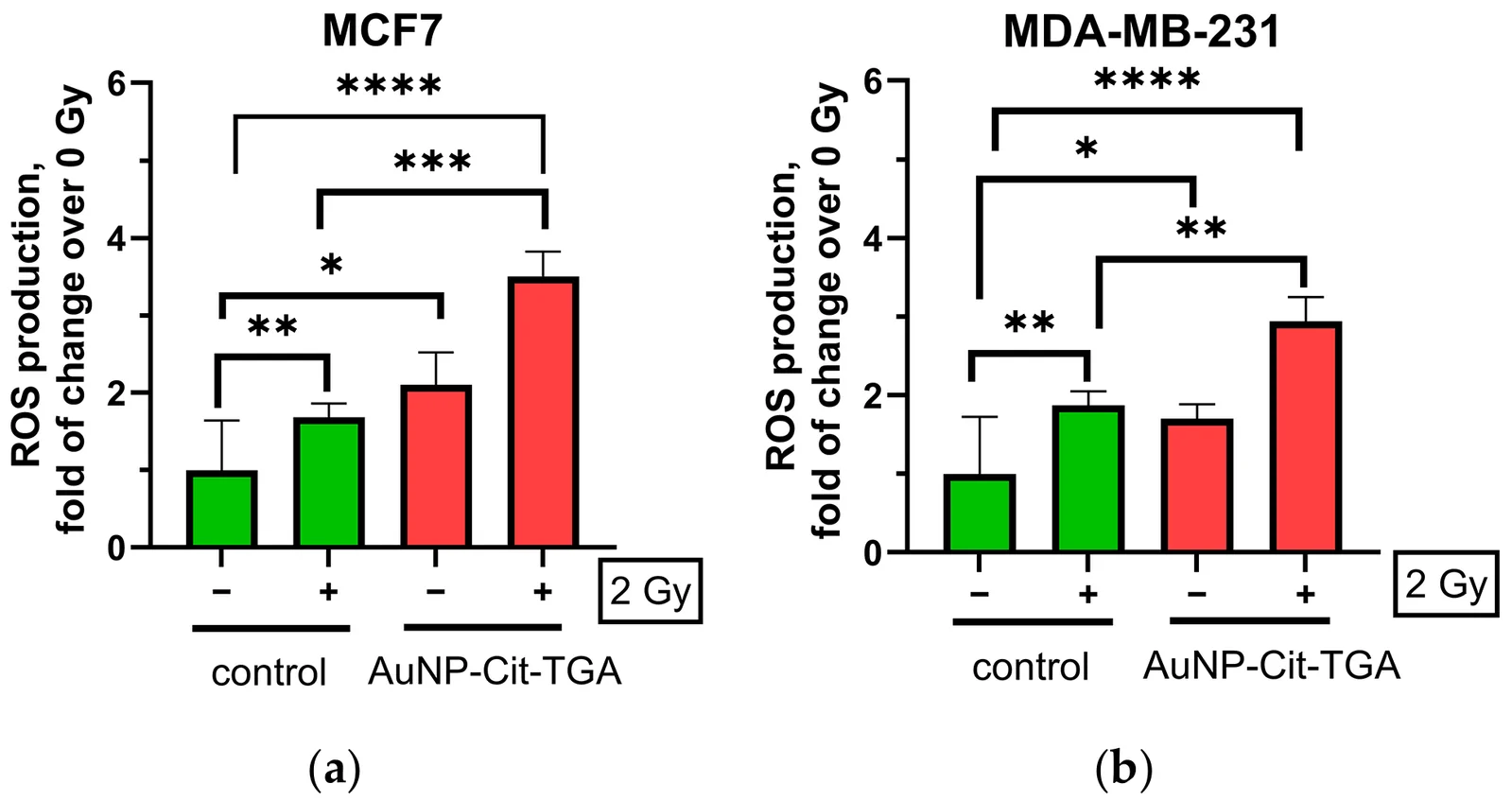
Radiation-induced ROS generation in (**a**) MCF7 and (**b**) MDA-MB-231 cells pre-treated with NPs. Cells were co-incubated with NPs for 1 h before irradiation at a final concentration of 0.5 μg/mL. ROS production was assessed by quantification of DCFH fluorescence at 24 h after no (0 Gy) or 2 Gy irradiation. Data represented as fold of change over non-irradiated controls without NPs. Data are means ± SD from 4 independent experiments. * *p* < 0.05, ** *p* < 0.01, *** *p* < 0.001, **** *p* < 0.0001 by Mann–Whitney U test.

**Figure 7:**
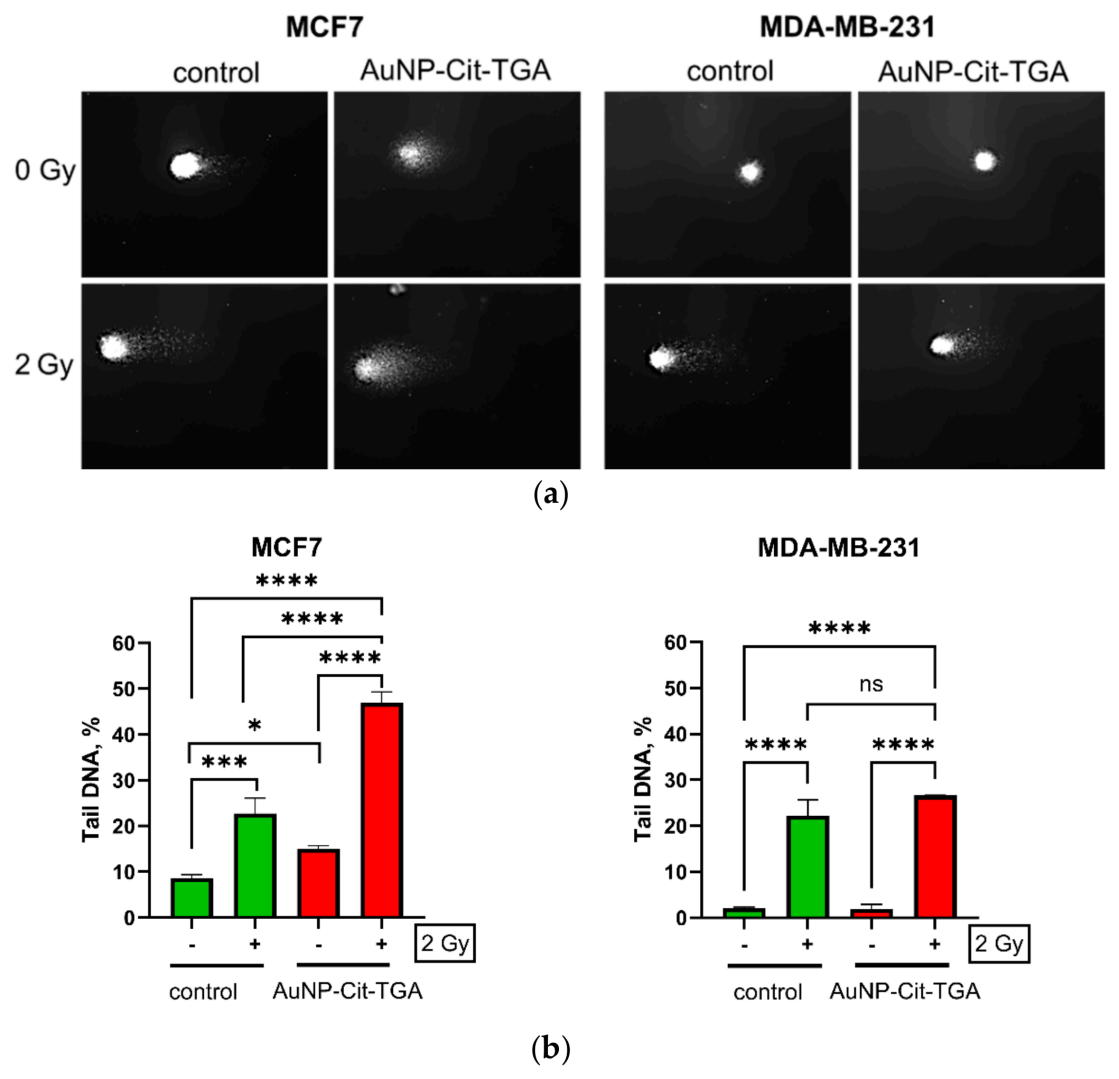
Single-stranded DNA breaks (SSBs) in MCF7 and MDA-MB-231 cells in response to radiation and pre-treatment with NPs. The cells were pre-incubated for 1 h with 0.5 µg/mL of AuNP-Cit-TGA NPs. (**a**) Representative images of the Comet assay, objective 40 x, and (**b**) the percentage of tail DNA (%) was assessed immediately after either no (-) or 2 Gy (+) irradiation. Data are means ± SD from 4 independent experiments. * *p* < 0.05, *** *p* < 0.001, **** *p* < 0.0001 by Mann–Whitney U test.

**Figure 8:**
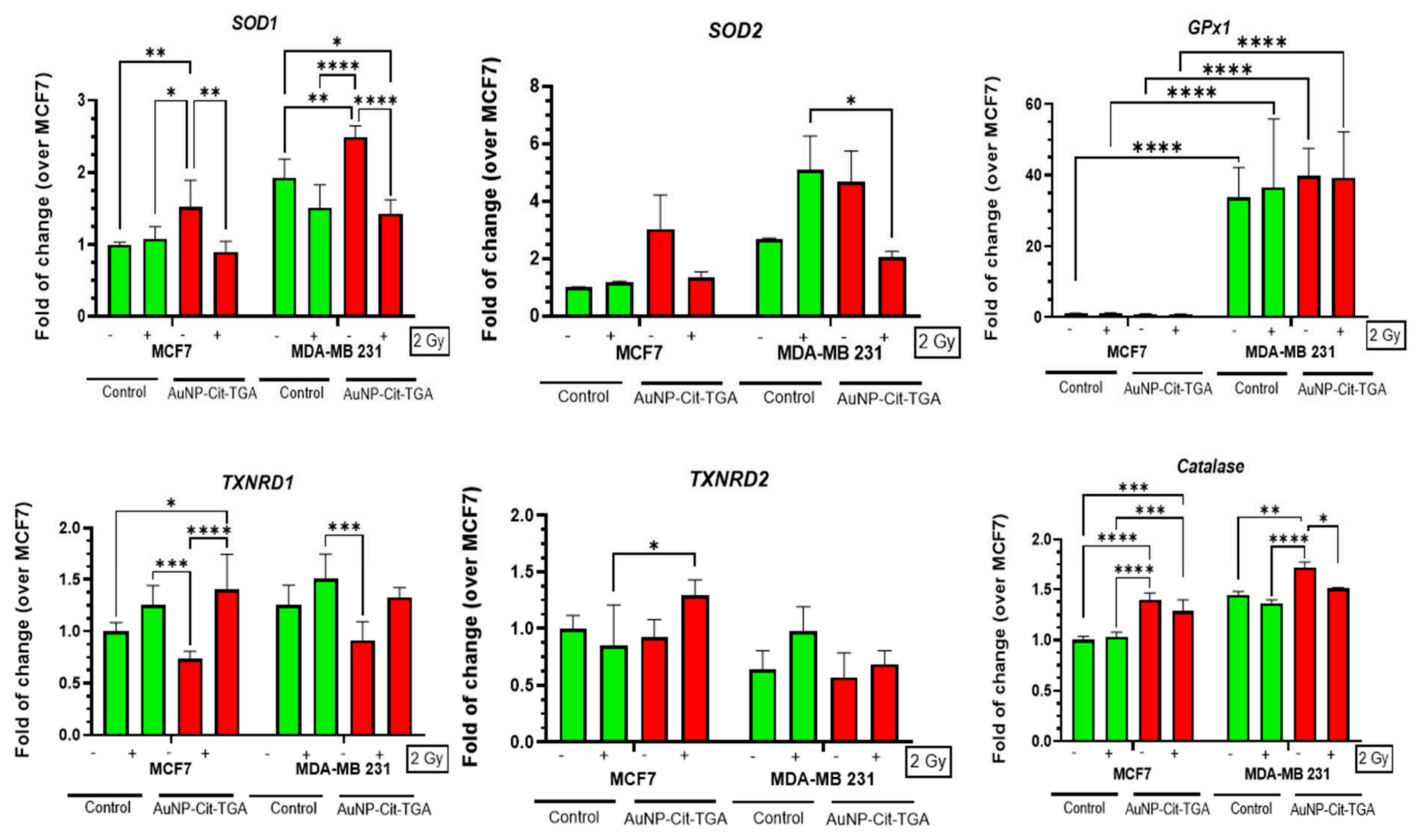
PCR analysis of mRNA of *SOD1*, *SOD2*, *GPx1*, *TXNRD*1, *TXNRD2* and *CAT* gene expression levels following 24 h of either no (-) or 2 Gy (+) irradiation. Data are means ± SD from 4 independent experiments. * *p* < 0.05, ** *p* < 0.01, *** *p* < 0.001, **** *p* < 0.0001 by Mann–Whitney U test.

**Figure 9:**
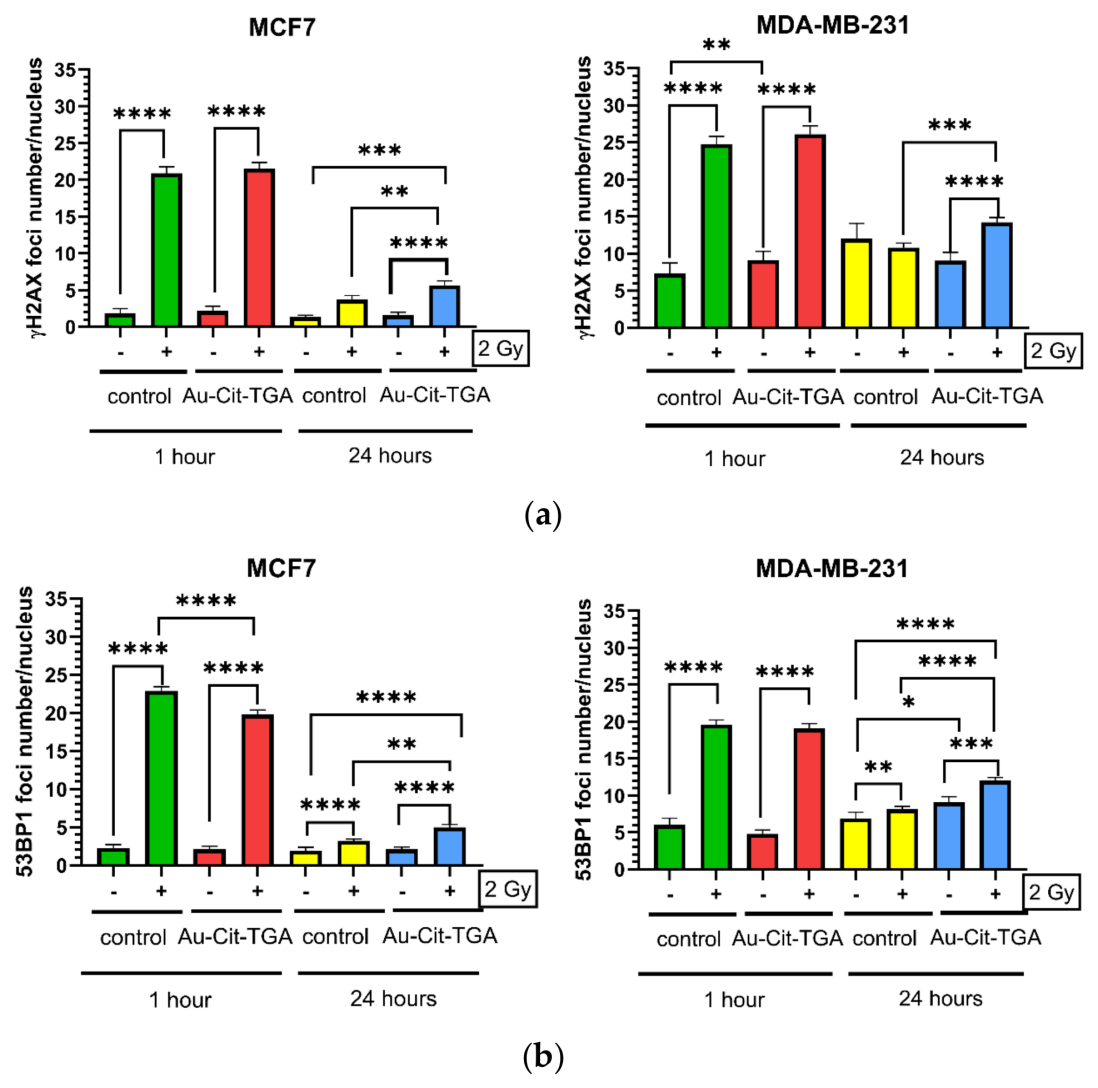
DNA damage response of BC cells. The number of (**a**) γH2AX and (**b**) 53BP1 foci were assessed at 1 h and 24 h after no (-) or 2 Gy (+) irradiation of cells that were pre-incubated either without (control) or with 0.5 µg/mL of NPs. Data are means ± SD from 4 independent experiments. * *p* < 0.05, ** *p* < 0.01, *** *p* < 0.001, **** *p* < 0.0001 by Mann–Whitney U test.

**Figure 10:**
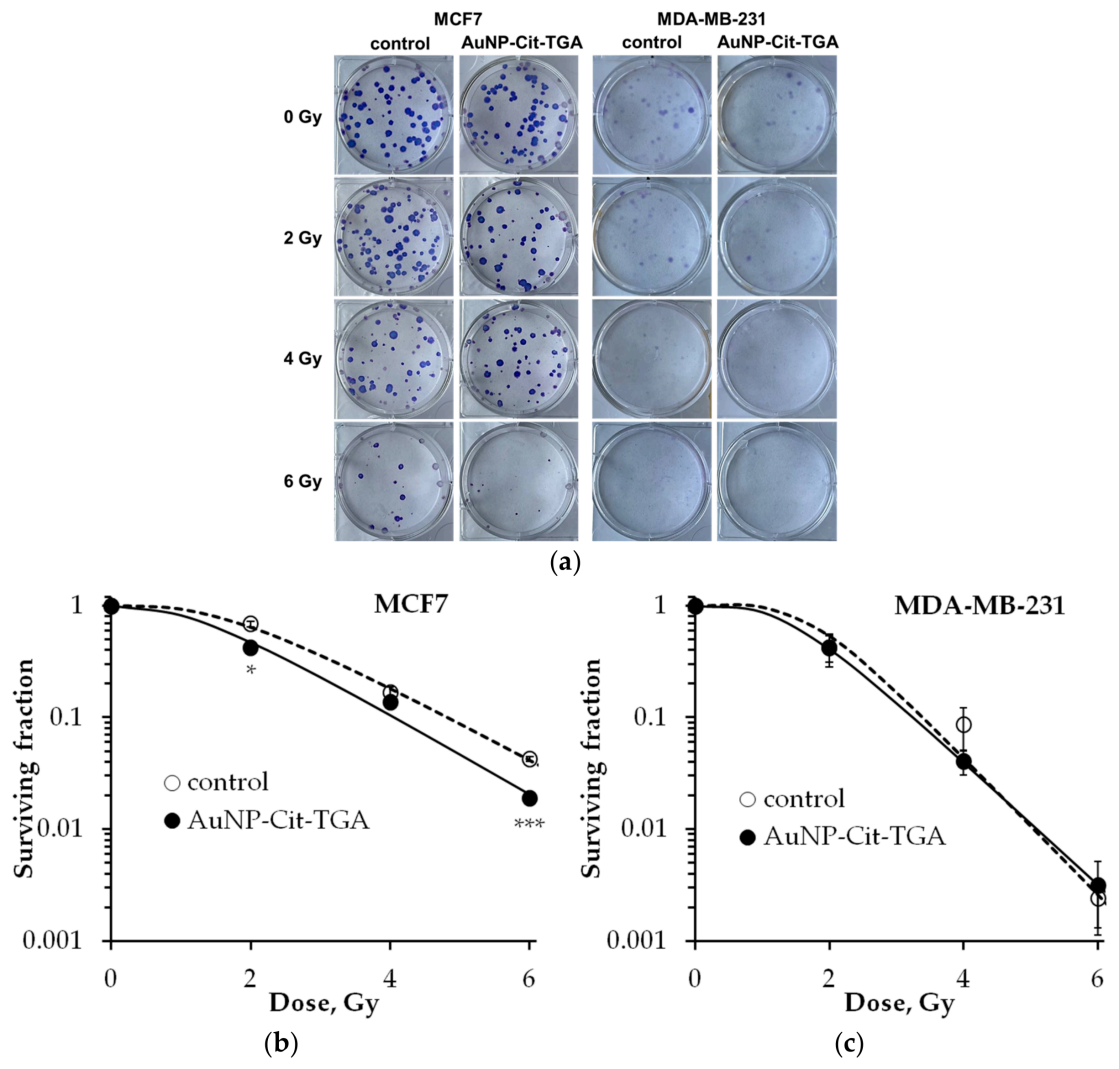
Radiosensitivity of MCF7 and MDA-MB-231 cells was estimated using clonogenic assay. (**a**) Representative photographs of colonies stained with crystal violet after 21 days of culture. Survival fraction curves of (**b**) MCF7 and (**c**) MDA-MB-231 cells. Data are means ± SD from three independent experiments. * *p* < 0.05, *** *p* < 0.001 by *t*-test.

**Table 1:** Mean hemolysis values for NPs, %.

	Concentration of AuNP-Cit-TGA, μg/mL					
Donor #	0.5	5	25	50	100	200
1	0.05	0.00	0.03	0.11	0.43	0.81
2	0.29	0.00	0.00	0.00	0.38	1.58
3	0.30	0.56	0.16	0.15	0.11	0.12
4	0.31	0.49	0.44	0.86	0.07	0.00

**Table 2:** The alpha and beta values for each cell line, calculated from the radiation survival curves using the linear quadratic model of fitting.

Cell Line	α	β	α/β
MCF7	0.18	0.06	2.95
MCF7+NPs	0.26	0.07	3.88
MDA-MB-231	0.04	0.16	0.24
MDA-MB-231+NPs	0.36	0.10	3.45

## Data Availability

The data presented in this study are available on request from the corresponding author.
